# Prevalence, awareness, treatment, and risk factor control of high atherosclerotic cardiovascular disease risk in Guangzhou, China

**DOI:** 10.3389/fcvm.2023.1092058

**Published:** 2023-07-14

**Authors:** Hui Liu, Weiquan Lin, Kexin Tu, Qin Zhou, Chang Wang, Minying Sun, Yaohui Li, Xiangyi Liu, Guozhen Lin, Sidong Li, Wei Bao

**Affiliations:** ^1^ Department of Basic Public Health, Center for Disease Control and Prevention of Guangzhou, Guangzhou, China; ^2^ Institute of Public Health, Guangzhou Medical University & Guangzhou Center for Disease Control and Prevention, Guangzhou, China; ^3^ Department of Epidemiology, School of Public Health, Sun Yat-sen University, Guangzhou, China; ^4^ Institute of Public Health Sciences, Division of Life Sciences and Medicine, University of Science and Technology of China, Hefei, China

**Keywords:** cardiovascular risk, prevalence, awareness, treatment, Guangzhou, risk assessment & management, ASCVD risk assessment

## Abstract

**Background:**

Identifying individuals at high risk of atherosclerotic cardiovascular disease (ASCVD) and implementing targeted prevention strategies might be the key to reducing the heavy disease burden in China. This study aimed to evaluate the prevalence, awareness, treatment, and risk factor control among individuals with high 10-year ASCVD risk in Guangzhou, China.

**Methods:**

This study included 15,165 adults (aged 18 years and older) from 138 urban and rural communities in the 2018 survey of China Chronic Disease and Risk Factors Surveillance in Guangzhou. 10-year ASCVD risk was estimated using the risk assessment models recommended in the Chinese Guideline for the Prevention of Cardiovascular Disease 2017. The prevalence, awareness, treatment, and risk factor control of high ASCVD risk (defined as 10-year risk ≥10%) were examined.

**Results:**

Among the study population, the weighted proportion of men was 51.9%, and the mean age was 41.27 ± 0.52 years. The overall standardized prevalence of high 10-year ASCVD risk was 13.8% (95% CI, 12.4%–15.3%). The awareness rates for hypertension, diabetes, and hyperlipidemia were 48.0% (95% CI, 42.8%–53.4%), 48.3% (95% CI, 43.0%–53.7%), and 17.9% (95% CI, 14.4%–22.1%) among those with corresponding risk factors. The proportions of drug use in prevention were relatively low in primary prevention, with the rates of using BP-lowering, glucose-lowering, lipid-lowering, and aspirin being 37.7% (95% CI, 32.8%–42.8%), 41.4% (95% CI, 35.8%–47.3%), 6.7% (95% CI, 4.5%–10.0%), and 1.0% (95% CI, 0.6%–1.8%), respectively. As for risk factor control, only 29.3% (95% CI, 25.7%–33.2%), 16.8% (95% CI, 15.0%–18.6%), and 36.0% (95% CI, 31.1%–41.2%) of individuals with high ASCVD risk had ideal levels of blood pressure, LDL-C, and body weight.

**Conclusion:**

The estimated prevalence of 10-year high ASCVD risk was high in Guangzhou, while the rates of treatment and risk factor control in primary prevention were still far from optimal, especially for lipid management. These findings suggested that substantial improvement in ASCVD prevention is needed in this population.

## Background

Cardiovascular disease (CVD) is the leading cause of death in China, accounting for over 40% of deaths in 2019 (1, 2). It is estimated that eliminating common modifiable risk factors could effectively prevent approximately three-fifths of incident CVD and mortality in China, especially for those metabolic risk factors (3, 4). Current guidelines in the prevention of atherosclerotic cardiovascular disease (ASCVD) have emphasized that the intensity of prevention efforts should be based on the absolute risk evaluation for individuals, and more intensive lifestyle modifications and evidence-based preventive medications should be recommended for those with higher absolute risk for ASCVD.

Identifying individuals at high risk of CVD and conducting targeted prevention strategies might be the key to reducing the considerably heavy burden of CVD in China. One prior national project on CVD screening and management suggested that almost 1 in 10 Chinese adults had a high risk for CVD in China, and the proportions of treatment with preventive pharmacotherapy were relatively low (0.6% for statins; 2.4% for aspirin) (5, 6). However, previous studies have well documented that the distribution of common risk factors as well as the burden of CVD varied dramatically across the country (7, 8). Some regional and community-level studies also found large variations in the proportions of individuals with high ASCVD risk (from 8.5% to 44.9%) (9–13). These disparities could be related to population characteristics, regional variations, and methods for estimating 10-year risk. Thus, accurate regional estimates of the prevalence and characteristics of high ASCVD risk may be informative for understanding the disease burden and developing context-specific strategies in different regions.

In 2017, the Chinese Guideline for the Prevention of Cardiovascular Disease recommended a new risk prediction chart developed for risk assessment in the Chinese population, which calculated the predicted 10-year risk for CVD on the basis of age, sex, smoking status, systolic blood pressure, the presence of diabetes, and lipid levels (14, 15). In this study, we applied the risk assessment models recommended by Chinese guidelines to estimate the prevalence of individuals with high ASCVD risk in Guangzhou using the China Chronic Disease and Risk Factors Surveillance (CCDRFS) data collected in the 2018 survey cycle. Besides, we also documented the awareness, treatment, and control of risk factors among those high-risk populations.

## Methods

### Study design and participants

Details of the study design and data resource profile of the CCDRFS have been reported previously (16–18). To be brief, the CCDRFS-Guangzhou study is a representative cross-sectional survey assessing the prevalence of non-communicable diseases and related risk factors, which recruited permanent residents aged over 18 years from all 11 districts in Guangzhou. Participants were selected based on a multistage stratified sampling framework with probability proportional to size to ensure the representativeness of the study population (Supplementary Figure S1). First, we randomly selected 1/3 of the communities in districts with ≥15 communities and five communities in those with <15 communities. The number of communities selected was proportional to the number of primary medical and healthcare institutions within each district. In all 11 districts of Guangzhou, we included a total of 69 community/township healthcare centers and then randomly selected two residential committees/villages from each community/township. Further, a total of 5,000 households were randomly selected from the 138 committees/villages using cluster sampling, and all family members aged 18 years and older who have been living at their current residence for more than six months were enrolled. Finally, a total of 15,727 residents were enrolled. After excluding participants with missing information on demographics or key variables used for predicting 10-year ASCVD risk (*n* = 562), a total of 15,165 participants aged 18 years or older were included in the current analysis. When comparing the baseline characteristics between those included and excluded in the current analysis, we found that those excluded due to missing information were more likely to be men and younger (Supplementary Table S1). The study protocol was approved by the ethical committee of the Guangzhou Center for Disease Control and Prevention. All participants provided written informed consent.

### Data collection and variables

Face-to-face interviews were conducted to collect information on demographic characteristics (sex, age, education level, occupation, marital status, and medical insurance status), health-related lifestyle behaviors (smoking, drinking, physical activity), and medical history by trained staffs during home visits using standardized protocols (16). Physical measurements (blood pressure, height, weight, waist and hip circumferences) and biochemical samples were centrally collected in community health centers. Fasting blood samples were drawn and transported to Guangzhou Center for Disease Control and Prevention within six hours of collection. Samples were analyzed for total cholesterol (TC), low-density lipoprotein cholesterol (LDL-C), high-density lipoprotein cholesterol (HDL-C), triglycerides (TG), blood glucose and homocysteine (HCY) were measured using enzyme colorimetry (Roche COBAS C501 automatic biochemical analyzer). Uric acid creatinine and urea nitrogen were measured using colorimetry (Roche c702 automatic biochemical analyzer).

Based on the status of smoking self-reported at the time of survey and referred to the definition of Global Adults Tobacco Survey (19), individuals were categorized into three groups: never, former, and current smokers. Respondents were asked whether they had ever drunk any kind of alcoholic beverage over the past 12 months; if yes, they were asked to provide information on frequency, types and quantity consumed in a typical day. Overdrinking was defined as consuming alcohol at least three times a week for more than six months. Sleep debt was defined as a sleeping duration of less than 7 h within 24 h. Physical activity was collected using the short-form International Physical Activity Questionnaire (20), and individuals did moderate or vigorous physical activity <150 min per week were considered as physical inactivity. Hypertension was defined as a baseline systolic and diastolic blood pressure of at least 140/90 mmHg, self-reported history of hypertension. Diabetes was defined as fasting glucose greater than or equal to 7 mmol/L or self-reported history of diabetes. Hyperlipidemia was defined as TC than or equal to 6.2 mmol/L, LDL-C than or equal to 4.1 mmol/L, TG than or equal to 2.3 mmol/L, HDL-C than or equal to 1.0 mmol/L, or self-reported history of hyperlipidemias. Information on medication use included a prescription of antiplatelet therapy, blood pressure control, lipid-lowering, or glucose control during the previous two weeks. Awareness and treatment of common risk factors (hypertension, diabetes, and hyperlipidemia) were assessed in participants with corresponding risk factors. Besides, we evaluated whether participants had their risk factors controlled as BP < 130/80 mm Hg; LDL-C < 2.6 mmol/L; not a current smoker; BMI of 18.5–24.0 kg/m^2^; and achieving physical activity targets (≥150 min of moderate-intensity physical activity per week or ≥75 min of high-intensity physical activity per week) (21).

### Cardiovascular risk assessment

Ten-year ASCVD (including nonfatal myocardial infarction, fatal or nonfatal stroke, and CVD death) risk was evaluated using the risk assessment model recommended in the Chinese Guideline for the Prevention of Cardiovascular Disease (14). The risk prediction chart was developed from a large multi-provincial cohort, which has been suggested to show good discrimination and calibration when predicting 10-year cardiovascular events, coronary heart diseases, and lifetime CVD risk (22–24). The Chinese guideline suggests evaluating the ASCVD risk hierarchically and categorizing individuals into different risk stratifications as low, moderate, high, and extremely high levels. Individuals with a prior history of CVD were categorized as extremely high risk. Individuals who met at least one of the following criteria were directly categized as high risk: (1) diabetic patients aged 40 years or older; (2) LDL-C ≥ 4.9 mmol/L (190 mg/dl) or TC ≥ 7.2 mmol/L (280 mg/dl); (3) SBP ≥ 180 mmHg or DBP ≥ 110 mmHg; (4) smoking ≥30 cigarettes per day. For the remaining people, the predicted 10-year risk for ASCVD was calculated based on age, sex, smoking status, blood pressure, and lipids levels (TC, LDL-C, HDL-C) by using the risk prediction charts. Detailed information for the risk assessment flowchart is listed in Supplementary Figure S2. Individuals with a predicted 10-year risk for ASCVD of <5%, 5%–9%, and ≥10% are categorized as low, moderate, and high risk, respectively. Similar to the guidelines from the American Heart Association and American College of Cardiology (25), the Chinese guideline recommends that primary prevention of ASCVD should be based on risk assessment tools. In this study, we categorized participants into low-to-moderate risk, high risk, and extremely high risk (those with established CVD). CVD was defined as a history of stroke, myocardial infarction, angina pectoris, coronary artery bypass grafting, or percutaneous coronary intervention.

### Statistical analysis

In this study, we reported the estimates of the prevalence of high ASCVD risk; rates of awareness, treatment, and control of related risk factors. Sampling weights were used in the analyses to improve the representativeness. The weights included sampling weights, non-response weights, and population weights (using the 7th population census of Guangzhou in 2020). The weighted adjusted percentages and 95% confidence intervals (95% CI) were used. The comparison of the ratios of different population characteristics was performed using the corrected Rao-Scott *χ*^2^ test based on a complex sampling design. Besides, we also assessed the association between participant characteristics and predicted high 10-year ASCVD among those without established CVD using logistic regression based on a complex sampling design. We further explored if the associations varied by sex and tested the interaction using multiplicative models by including the product terms. To compare the rates of awareness, treatment, and control between key demographic subgroups, we also applied logistic regression models with a complex sampling design adjusting for age, sex, urban or rural residency, and education levels. Statistical analysis was performed using SPSS V.21.0. Two-sided *P*-value <0.05 was considered statistically significant.

## Results

### Characteristics of participants

Among 15,165 participants included in the current analysis, 289 had established CVD, 3,075 had high 10-year ASCVD risk, and 11,801 had low-to-moderate 10-year risk. The weighted proportion of men was 51.9%, and the mean age was 41.27 ± 0.52 years (Table 1). Compared to those with low-to-moderate risk, participants with CVD and predicted 10-year ASCVD risk ≥10% were more likely to be men, with older age, and less educated, and they were also more prevalent to have metabolic risk factors (including obesity, hypertension, abnormal HCY, and renal function damage). Whereas, participants with high 10-year risk were more frequently to smoke and drink alcohol currently, have sleep debt, expose to second-hand smoke, and have diabetes and dyslipidemia than those with low-to-moderate risk or with established CVD.

**Table 1:** Baseline characteristics of study population.^a^

Characteristic	Overall (*N* = 15,165)	Low or moderate 10-year risk of ASCVD (*N* = 11,801)	High 10-year risk of ASCVD (*N* = 3,075)	Established CVD (*N* = 289)
Sex, *n* (%)				
Men	5,962 (51.9)	4,122 (49.3)	1,662 (67.2)	178 (68.9)
Women	9,203 (48.1)	7,679 (50.7)	1,413 (32.8)	111 (31.1)
Mean age (SD), y	41.27 (0.52)	39.05 (0.53)	53.50 (0.59)	63.95 (1.34)
Age, year, *n* (%)				
18 < 25	612 (12.7)	601 (14.6)	11 (1.7)	0 (0.0)
25 < 35	2,133 (28.1)	2,056 (31.7)	76 (7.8)	1 (0.1)
35 < 45	2,584 (22.3)	2,387 (24.0)	189 (12.8)	8 (7.6)
45 < 55	3,268 (19.1)	2,552 (16.8)	688 (33.9)	28 (17.5)
55 < 65	3,851 (10.3)	2,574 (7.9)	1,181 (24.5)	96 (25.4)
≥65	2,717 (7.5)	1,631 (5.1)	930 (19.4)	156 (49.4)
Urban, *n* (%)	10,739 (68.3)	8,471 (68.8)	2,044 (64.8)	224 (79.6)
Education level, *n* (%)				
Primary school or less	3,740 (13.7)	2,575 (11.5)	1,075 (27.0)	90 (25.8)
Middle school/High school	8,160 (51.6)	6,278 (50.3)	1,719 (58.8)	163 (57.8)
College or higher	3,265 (34.7)	2,948 (38.2)	281 (14.2)	36 (16.4)
BMI, *n* (%)				
<24	8,516 (58.6)	7,038 (61.8)	1,346 (39.3)	132 (43.5)
24 < 28	4,882 (29.7)	3,611 (28.1)	1,164 (38.6)	107 (40.6)
≥28	1,767 (11.8)	1,152 (10.1)	565 (22.1)	50 (15.9)
Abdominal obesity, *n* (%)	4,716 (25.9)	3,173 (22.6)	1,402 (45.2)	141 (49.4)
Hypertension, *n* (%)	4,767 (20.3)	2,617 (14.0)	1,939 (56.5)	211 (70.2)
Diabetes, *n* (%)	1,750 (7.4)	92 (1.3)	1,573 (43.9)	85 (24.4)
Dyslipidemia, *n* (%)	5,603 (32.4)	3,480 (26.6)	1,975 (67.0)	148 (46.7)
Current smoking, *n* (%)	2,544 (21.2)	1,409 (17.5)	1,073 (44.2)	62 (23.2)
Current drinking, *n* (%)	4,342 (38.0)	3,345 (37.4)	997 (42.0)	81 (25.8)
Over drinking, *n* (%)	433 (3.4)	237 (2.5)	190 (9.3)	6 (1.3)
Sleep-debt, *n* (%)	2,645 (14.9)	1,918 (13.7)	671 (22.3)	56 (15.6)
Second-hand smoke exposure, *n* (%)	9,162 (63.7)	7,008 (62.8)	1,995 (69.6)	159 (59.1)
abnormal HCY, *n* (%)^b^	2,429 (11.0)	1,470 (8.6)	843 (23.7)	116 (42.1)
Renal function damage, *n* (%)^c^	7,551 (50.5)	5,579 (49.6)	1,794 (55.6)	178 (54.1)
History of hemorrhage stroke, *n* (%)	25 (0.1)	0 (0.0)	0 (0.0)	25 (5.9)
CVD family history, *n* (%)	1,492 (9.4)	1,122 (9.3)	312 (9.2)	58 (20.7)

ASCVD, atherosclerotic cardiovascular disease; CIs, confidence intervals; TG: triglycerides; BMI: body mass index; HCY: homocysteine; CVD, cardiovascular diseases.

^a^ No. of participants was the unweighted number of subcategories denominator; the percentages were weighted.

^b^ Abnormal HCY means homocysteine levels <5 µmol/L or >15 µmol/L.

^c^ Renal function damage means abnormalities in any of the three laboratory tests: blood uric acid, blood creatinine and blood urea nitrogen (BUN).

### Prevalence of individuals with high ASCVD risk and established CVD

The overall standardized prevalence of high 10-year ASCVD risk was 13.8% (95% CI, 12.4%–15.3%), and 0.9% (95% CI, 0.7%–1.2%) had prevalent CVD (Table 2). The prevalence of high predicted 10-year ASCVD risk was higher in men (weighted prevalence, 17.8; 95% CI, 15.1%–21.0%) compared to women (9.4%; 95% CI, 8.1%–10.8%), and significantly increased by age, with 35.6% (95% CI, 32.9%–38.4%) of people aged 65 years or older predicted to be with high risk. Moreover, the prevalence of high 10-year ASCVD risk was similar between urban and rural areas (urban: 13.1%; 95% CI, 11.2%–15.2% vs. rural: 15.3%; 95% CI, 13.3%–17.6%), but the prevalence was significantly higher in those with primary education (27.1; 95% CI, 23.9%–30.5%) than those with high education levels (5.6%; 95% CI, 4.5%–7.1%). The variations in the prevalence of CVD by key demographic characteristics showed similar patterns to those in high ASCVD risk. Among those who were estimated to have a moderate risk of 10-year ASCVD and aged <55 years, 46.3% (95% CI, 39.9%–52.9%) were assessed to have a high lifetime cardiovascular risk (Supplementary Table S2). The prevalence of high lifetime ASCVD risk was higher among men, those younger than 35 years, and those with high levels of education.

**Table 2:** Age and sex adjusted prevalence of population with high 10-year ASCVD risk and with established CVD.^a^

Characteristic	Low or moderate 10-year risk of ASCVD (*N* = 11,801)		High 10-year risk of ASCVD (*N* = 3,075)		Established CVD (*N* = 289)		*P*-value
	*n*	% (95%CIs)	*n*	% (95%CIs)	*n*	%(95%CIs)	
Overall	11,801	85.3 (83.7–86.8)	3,075	13.8 (12.4–15.3)	289	0.9 (0.7–1.2)	
Sex							<0.001
Men	4,122	80.9 (77.7–83.8)	1,662	17.8 (15.1–21.0)	178	1.2 (0.9–1.6)	
Women	7,679	90.0 (88.5–91.3)	1,413	9.4 (8.1–10.8)	111	0.6 (0.4–0.9)	
Age, year							<0.001
18 < 25	601	98.1 (96.1–99.1)	11	1.9 (0.9–3.9)	0	–	
25 < 35	2,056	96.2 (94.8–97.2)	76	3.8 (2.8–5.2)	1	0.0 (0.0–0.0)	
35 < 45	2,387	91.8 (90.0–93.3)	189	7.9 (6.4–9.7)	8	0.3 (0.1–0.7)	
45 < 55	2,552	74.7 (72.5–76.9)	688	24.4 (22.2–26.8)	28	0.8 (0.5–1.5)	
55 < 65	2,574	65.0 (60.9–68.9)	1,181	32.7 (28.8–36.9)	96	2.3 (1.6–3.2)	
≥65	1,631	58.3 (55.3–61.2)	930	35.6 (32.9–38.4)	156	6.1 (4.4–8.3)	
Residence							0.07
Urban	8,471	85.9 (83.7–87.8)	2,044	13.1 (11.2–15.2)	224	1.1 (0.8–1.5)	
Rural	3,330	84.1 (81.7–86.2)	1,031	15.3 (13.3–17.6)	65	0.6 (0.4–0.8)	
Education level							<0.001
Primary school or less	2,575	71.2 (67.5–74.6)	1,075	27.1 (23.9–30.5)	90	1.7 (1.2–2.4)	
Middle school/High school	6,278	83.3 (81.2–85.1)	1,719	15.7 (13.9–17.7)	163	1.0 (0.7–1.5)	
College or higher	2,948	93.9 (92.4–95.2)	281	5.6 (4.5–7.1)	36	0.4 (0.2–0.8)	

ASCVD, atherosclerotic cardiovascular disease; CIs, confidence intervals; TG: triglycerides; BMI: body mass index; HCY: homocysteine; CVD, cardiovascular diseases.

^a^ No. of participants was the unweighted number of subcategories denominator; the percentages were weighted.

### Factors associated with high predicted ASCVD risk among individuals without CVD

Table 3 shows the association between individual risk factors and high ASCVD risk among participants without established CVD. Older age, rural areas, higher BMI, abdominal obesity, overdrinking, sleep debt second-hand smoke exposure, and abnormal HCY were significantly associated with a predicted 10-year risk higher than 10%. When stratified by sex, location of residence, education level, overdrinking and second-hand smoke exposure had a stronger association with predicted ASCVD risk in men. By contrast, age and renal function damage had more pronounced associations among women.

**Table 3:** The association of different risk factors with ASCVD by logistic regression analysis among individuals without established CVD and stratified by sex.^a^

Characteristic	Overall		Men		Women		*P*_two-way interaction_ ^b^	*P*_three-way interaction_ ^c^
	OR (95%CIs)	*P*-value	OR (95%CIs)	*P*-value	OR (95%CIs)	*P*-value		
Age, year							0.002	−
18 < 25	1.00		1.00		1.00			
25 < 35	1.71 (0.82–3.57)	0.147	1.46 (0.63–3.36)	0.370	2.96 (0.82–10.70)	0.097		
35 < 45	3.65 (1.64–8.13)	0.002	3.71 (1.45–9.50)	0.007	4.10 (1.20–13.97)	0.025		
45 < 55	12.63 (6.02–26.52)	<0.001	14.24 (5.95–34.10)	<0.001	15.71 (4.80–51.43)	<0.001		
55 < 65	19.89 (9.23–42.86)	<0.001	18.12 (7.31–44.92)	<0.001	39.78 (12.14–130.41)	<0.001		
≥65	24.43 (11.27–52.99)	<0.001	19.22 (7.76–47.58)	<0.001	58.15 (17.32–195.21)	<0.001		
Residence							0.009	0.99
Urban	1.00		1.00		1.00			
Rural	1.25 (1.05–1.48)	0.014	1.33 (0.99–1.77)	0.055	0.77 (0.57–1.03)	0.080		
Education level							0.03	1.00
Primary school or less	1.00		1.00		1.00			
Middle school/High school	1.18 (0.95–1.47)	0.124	0.84 (0.67–1.05)	0.119	1.02 (0.78–1.32)	0.904		
College or higher	0.84 (0.60–1.18)	0.308	0.48 (0.32–0.71)	0.000	1.08 (0.69–1.69)	0.741		
BMI							0.56	0.96
<24	1.00		1.00		1.00			
24 < 28	1.39 (1.15–1.68)	0.001	1.38 (1.09–1.76)	0.009	1.24 (1.02–1.51)	0.028		
≥28	2.26 (1.74–2.95)	<0.001	2.38 (1.63–3.46)	<0.001	1.92 (1.49–2.47)	<0.001		
Abdominal obesity	1.35 (1.14–1.60)	0.001	1.35 (1.07–1.69)	0.012	1.41 (1.18–1.68)	<0.001	0.79	0.53
Over drinking	2.90 (1.94–4.33)	<0.001	2.29 (1.49–3.50)	<0.001	0.34 (0.10–1.19)	0.091	0.003	0.91
Sleep-debt	1.27 (1.08–1.51)	0.006	1.49 (1.13–1.97)	0.006	0.97 (0.73–1.28)	0.814	0.08	0.06
Second-hand smoke exposure	1.49 (1.25–1.78)	<0.001	1.56 (1.22–2.00)	0.001	1.14 (0.92–1.40)	0.223	0.003	0.16
Abnormal HCY	1.29 (1.10–1.52)	0.002	1.03 (0.86–1.23)	0.780	0.94 (0.76–1.17)	0.592	0.12	0.99
Renal function damage	1.18 (0.97–1.45)	0.105	1.05 (0.79–1.39)	0.751	1.43 (1.23–1.66)	<0.001	0.04	0.05
CVD family history	1.06 (0.86–1.32)	0.572	1.23 (0.88–1.72)	0.225	0.93 (0.72–1.21)	0.588	0.46	1.00

ASCVD, atherosclerotic cardiovascular disease; CVD, cardiovascular diseases; established CVD, including stroke, myocardial infarction, angina pectoris, heart bypass surgery, heart stent surgery; SE, standard error; OR, odds ratio; CIs, confidence intervals; BMI: body mass index; TG: triglycerides.

^a^ Using unconditional logistic regression based on complex sampling designs. The model was adjusted for age, residence, education level, BMI (<24/24 < 28/ ≥ 28), abdominal obesity, over drinking, sleep-debt, second-hand smoke exposure, abnormal HCY, renal function damage, CVD family history.

^b^ The interaction between sex and the risk of ASCVD was evaluated by multiplicative models by including the product term in multivariate logistic regression to test the variations in the associations between each risk factor and high 10-year ASCVD risk among men and women.

^c^ The interaction between sex, age group, and associated risk factors was evaluated by multiplicative models by including the product term in multivariate logistic regression to test the variations in the associations between risk factors with high 10-year ASCVD risk among men and women differed by age groups.

### Awareness of hypertension, diabetes, and hyperlipidemia

The awareness rates of common cardiovascular risk factors were highest among those with established CVD, followed by those with high 10-year ASCVD risk, and lowest among those with low or moderate risk. Among participants with high 10-year risk, the awareness rates for hypertension, diabetes, and hyperlipidemia were 48.0% (95% CI, 42.8%–53.4%), 48.3% (95% CI, 43.0%–53.7%), and 17.9% (95% CI, 14.4%–22.1%), respectively (Table 4). The awareness rates of these three risk factors were higher in women compared with men among those without known CVD, whereas the awareness rates of hypertension and hyperlipidemia were higher in men than women among participants with established CVD. When stratified by location of residence, urban residents consistently had a higher proportion of risk factor awareness than those living in rural areas. After adjusting for those key demographic variables, the awareness rate of diabetes was significantly higher in women than men among those with low or moderate risk [odds ratio (OR), 5.75; 95% CI, 1.78–18.57]. Among those with high ASCVD risk, hypertension awareness was higher in women (OR, 1.67; 95% CI, 1.16–2.41) and diabetes awareness was higher in urban residents (OR, 2.12; 95% CI, 1.47–3.05). In terms of established CVD, the awareness rates of hypertension (OR, 4.36; 95% CI, 1.04–18.32) and hyperlipidemia (OR, 6.31; 95% CI, 1.43–27.76) were both significantly higher in urban residents.

**Table 4:** Awareness rate among participants with low or moderate ASCVD risk, those with high ASCVD risk, or those with established CVD^a^.

	Low or moderate 10-year risk of ASCVD [*N *=* *11,801]				High 10-year risk of ASCVD [*N *=* *3,075]				Established CVD[*N *=* *289]				*p* Value^c^
	*n*/*N*	% (95% CIs)	*OR* (95% CIs)^b^	*p* Value^b^	*n*/*N*	% (95% CIs)	*OR* (95% CIs)^b^	*p* Value^b^	*n*/*N*	% (95% CIs)	*OR* (95% CIs)^b^	*p* Value^b^	
Awareness hypertension (%)													
Overall	1,313/2,617	40.7 (36.6–45.0)			1,082/1,939	48.0 (42.8–53.4)			178/211	82.5 (69.6–90.7)			0.001
Sex				0.146				0.007				0.478	
Men	406/840	35.9 (30.0–42.2)	1 (Ref)		578/1,126	41.6 (36.4–46.9)	1 (Ref)		104/125	86.2 (73.8–93.3)	1 (Ref)		
Women	907/1,777	45.1 (40.6–49.6)	1.26 (0.92–1.72)		504/813	62.4 (55.8–68.7)	1.67 (1.16–2.41)		74/86	75.7 (57.8–87.6)	0.78 (0.38–1.58)		
Residence				0.470				0.081				0.045	
Urban	898/1,727	43.1 (37.6–48.8)	1.13 (0.80–1.59)		725/1,221	51.1 (43.1–59.2)	1.32 (0.97–1.80)		144/163	87.4 (68.3–95.7)	4.36 (1.04–18.32)		
Rural	415/890	36.3 (31.2–41.7)	1 (Ref)		357/718	43.0 (38.0–48.1)	1 (Ref)		34/48	62.6 (43.2–78.6)	1 (Ref)		
Awareness diabetes (%)													
Overall	30/92	25.6 (16.6–37.3)			878/1,573	48.3 (43.0–53.7)			67/85	80.5 (68.2–88.8)			0.007
Sex				0.004				0.195				0.453	
Men	8/42	11.5 (4.9–24.6)	1 (Ref)		372/698	43.3 (37.4–49.5)	1 (Ref)		40/53	79.6 (61.6–90.4)	1 (Ref)		
Women	22/50	42.8 (28.4–58.6)	5.75 (1.78–18.57)		506/875	54.8 (48.8–60.6)	1.22 (0.90–1.64)		27/32	82.7 (59.6–93.9)	2.05 (0.30–14.01)		
Residence				0.958				<0.001				0.747	
Urban	19/62	24.8 (13.9–40.1)	0.97 (0.29–3.23)		670/1,142	52.6 (46.6–58.6)	2.12 (1.47–3.05)		56/69	82.8 (68.2–91.6)	1.42 (0.16–12.56)		
Rural	11/30	26.9 (13.3–46.9)	1 (Ref)		208/431	35.8 (29.1–43.1)	1 (Ref)		11/16	70.8 (42.4–88.8)	1 (Ref)		
Awareness hyperlipidemia (%)													
Overall	632/3,480	12.8 (10.8–15.2)			437/1,975	17.9 (14.4–22.1)			97/148	65.7 (53.5–76.1)			<0.001
Sex				0.451				0.412				0.421	
Men	228/1,433	12.0 (9.8–14.6)	1 (Ref)		216/992	16.5 (12.8–21.0)	1 (Ref)		53/82	73.7 (58.8–84.6)	1 (Ref)		
Women	404/2,047	14.3 (11.5–17.7)	1.11 (0.84–1.47)		221/983	20.8 (15.8–26.8)	1.25 (0.73–2.16)		44/66	53.4 (36.8–69.3)	0.65 (0.22–1.89)		
Residence				0.664				0.071				0.016	
Urban	517/2,528	13.9 (11.1–17.2)	0.92 (0.63–1.34)		337/1,355	21.4 (16.6–27.1)	1.52 (0.96–2.41)		84/120	74.7 (61.6–84.4)	6.31 (1.43–27.76)		
Rural	115/952	10.6 (8.3–13.5)	1 (Ref)		100/620	11.4 (8.4–15.3)	1 (Ref)		13/28	28.8 (14.3–49.4)	1 (Ref)		

ASCVD, atherosclerotic cardiovascular disease; CVD, cardiovascular diseases; established CVD, including stroke, myocardial infarction, angina pectoris, heart bypass surgery, heart stent surgery; CIs, confidence intervals.

^a^ *n* represents the actual number of people surveyed, and the composition ratio is weighted according to the complex sampling design (%).

^b^ *P*-values and *OR* (95% CIs) were calculated in logistic regression models, in which the dependent variable was the awareness of hypertension, diabetes or hyperlipidemia, and the dependent variables included sex, residence, age, and education level.

^c^ *P*-value was calculated in logistic regression models, in which the dependent variable was the awareness of hypertension, diabetes or hyperlipidemia, and the dependent variables included three risk groups, sex, residence, age, and education level.

### Medication use and risk factor control in primary and secondary prevention

**Figure 1:**
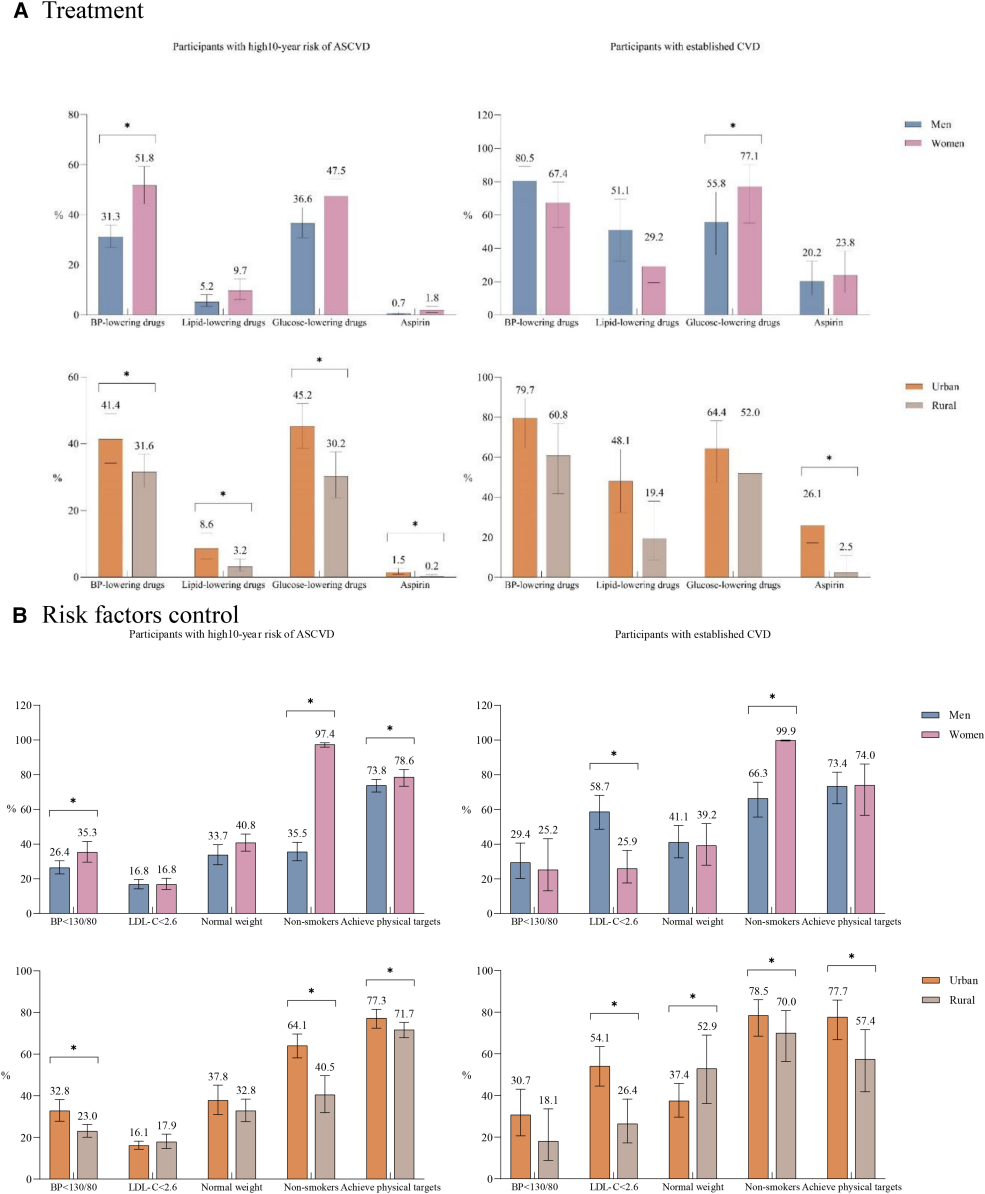
Treatment and risk factor control in primary prevention and secondary prevention. (**A**) Treatment. (**B**) Risk factors control. ASCVD, atherosclerotic cardiovascular disease; CVD, cardiovascular diseases; established CVD, including stroke, myocardial infarction, angina pectoris, heart bypass surgery, heart stent surgery. The use of BP lowering drugs, lipid-lowering drugs and glucose-lowering medications was calculated based on people with hypertension, diabetes and dyslipidemia respectively; the use of other medicine and the control of risk factors were based on the whole population. Normal weight means BMI ≥ 18.5–<24.0. Achieving physical activity targets means ≥150 min of moderate intensity exercise per week or ≥75 min of high intensity exercise per week. All ratios listed were weighted according to the complex sampling design. *P-value <0.05; *P*-values were calculated using logistic regression models with a complex sampling design adjusting for sex, residence, age, and education level.

The proportions of drug use in prevention were relatively low in primary prevention, with the rates of using BP-lowering, glucose-lowering, lipid-lowering, and aspirin being 37.7% (95% CI, 32.8%–42.8%), 41.4% (95% CI, 35.8%–47.3%), 6.7% (95% CI, 4.5%–10.0%), and 1.0% (95% CI, 0.6%–1.8%), respectively (Figure 1A and Supplementary Table S3). By contrast, the proportion of medication use was relatively higher in those with established CVD, which might be related to reverse causality. When stratified by sex, we found that women were more likely to receive BP lowering drugs (OR, 1.58; 95% CI, 1.05–2.38) in primary prevention and glucose-lowering medications in secondary prevention (OR, 5.62; 95% CI, 1.19–26.57). Moreover, the proportions of BP-lowering drugs, lipid-lowering drugs, glucose-lowering drugs, and aspirin were significantly higher in urban residents than those living in rural areas in primary prevention; and urban residents were also more likely to use aspirin in secondary prevention.

As for risk factor control, only 29.3% (95% CI, 25.7%–33.2%), 16.8% (95% CI, 15.0%–18.6%), and 36.0% (95% CI, 31.1%–41.2%) of individuals with high ASCVD risk could have their blood pressure, LDL-C, and body weight well controlled (Figure 1B). Women had higher proportions of blood achieving ideal blood pressure levels and weight status in primary prevention compared to men, whereas the prevalence of LDL-C < 2.6 mmol/L in secondary prevention was twice higher in men than that in women (58.7% vs. 25.9%). When stratified by location of residence, rural participants had significantly lower rates of BP control and non-smokers in primary prevention, as well as lipid control and achieving physical targets in secondary prevention.

## Discussion

In this regional representative cross-sectional survey, we found that one-seventh individuals had a high 10-year estimated high ASCVD risk in Guangzhou. Among those with metabolic risk factors, over half of individuals were not aware of their diagnosis of hypertension and diabetes, and more strikingly, only one-sixth of participants were aware of the dyslipidemia conditions. Moreover, the use of medical therapies, generally required to control metabolic risk factors, was also low in primary prevention, especially for aspirin and lipid-lowering medications.

We found that 13.8% (95% CI, 12.4%–15.3%) of adults were estimated to have a 10-year ASCVD risk higher than 10% in Guangzhou, which was almost consistent with those previous reports on the national scale (5, 9, 26). The CCDRFS in the 2010 survey cycle has found that the prevalence of high ASCVD risk in adults aged over 35 years old was 8.5% using a nationally representative sample (9), and another study using a convenience sample of 1.7 million adults aged 35–75 years between 2014 and 2017 reported that 9.5% of participants had a high risk for CVD (5). These two national study applied the same risk prediction chart as that used in our study (5, 9), which directly included age, sex, SBP, total cholesterol, HDL-C, smoking status, and diabetes mellitus. Whereas, another community-based survey suggested that 19.9% of adults aged 45 years older in China were categorized as high-risk population (26), and this study applied the China-PAR risk prediction model (27), which additionally included waist circumference, geographic region, urbanization, and family history of ASCVD for risk prediction. The difference in prevalence estimates could be related to the study time period, sampling strategies, population differences, and risk assessment methods. Whereas, to our knowledge, there is a lack of head-to-head comparison between different models for risk prediction in China and it remains unclear which model could provide better performance for risk prediction. In the current analysis, we applied the risk prediction chart recommended in the Chinese Guideline for the Prevention of Cardiovascular Disease, which was also commonly used in those previous studies evaluating the ASCVD risk burden in China and enabled indirect comparisons between different regions and time frames (5, 9, 14, 28). Within the past decade, a large body of literature has documented that the prevalence rates of hypertension (29, 30), diabetes (18), dyslipidemia (31, 32), and obesity (17) have significantly increased; and these four metabolic risk factors were major contributors to incident CVD in China (4). Meanwhile, the prevalence of lifestyle risk factors (such as smoking, physical inactivity, and unhealthy diet) is also widespread along with the rapid progress in urbanization and socioeconomic development in parallel (33), which might further trigger the deterioration of metabolic health in the Chinese population. Ultimately, the heavy burden of primary prevention for ASCVD might be further intensified in the following decades.

Previous estimates suggested that the rates of awareness, treatment, and control for these risk factors still remained inadequate in the general population in China, despite that identification and control of these metabolic diseases have been prioritized by the national government (18, 29–32). Cardiovascular risk assessment provides important information for guiding decision-making in primary prevention, and guidelines recommended the initiation of evidence-based preventive pharmacotherapy should be considered for those with high 10-year CVD risk (25, 34, 35). Nevertheless, we still found large gaps in treating and controlling hypertension, diabetes, and dyslipidemia among those high ASCVD risk populations, which was consistent with prior findings (5, 26). In particular, the largest gap between both detection and control was observed for dyslipidemia. In the current study, over two-thirds of high-risk individuals had been diagnosed with dyslipidemia, of whom only 17.9% were aware of their diagnosis and only 6.7% received lipid-lowering drugs. Of note, less than half of individuals with established CVD, for whom statins were recommended as first-line preventive medications irrespective of LDL-C levels, received lipid-lowering medications, was only 42.4%, and the use of statins was even lower. The low rates of detection and treatment might be related to the low rates of screening, difficulty in accessing health care, and unaffordability of medical services. Although statins have been low-cost and affordable after the health care reform in China, a national cross-sectional study suggested that less than half of primary care institutions in China stocked statins, and the rates were even lower in rural village clinics (32). Availability of statins and other lipid-lowering medications in primary care institutions might still be a large obstacle for lipid management in China.

We found substantial disparities in managing and controlling those risk factors between rural and urban communities, which could be related to the low affordability and availability of preventive medications and medical services among rural residents. Of note, in this study, we included participants from Guangzhou, the third most developed city in China, of which residents generally had better socioeconomic status than those from other parts of the country. Thus, the burden of high ASCVD in China might be even larger than we observed in this study. Moreover, despite men having a higher 10-year ASCVD risk, women consistently had higher awareness, treatment, and control of their risk factors in primary prevention, perhaps due to sex differences in health-seeking behavior (36). However, a reverse pattern was observed in the secondary prevention that women were less likely to receive lipid-lowering drugs and LDL-C control among those with established CVD. Similar findings have also been reported in a prior large multinational study that the patterns in differences between men and women in primary prevention were contrasting compared with secondary prevention (37). Previous studies that focused on differences in treatment following acute coronary syndrome events also suggested that women were undertreated (both evidence-based acute treatments during hospitalization and medications prescribed at discharge) in China (38). But a cross-sectional study conducted in China indicated that women with established CVD were less likely to receive guideline-recommended medications, and those at high risk were also less likely to achieve blood pressure, LDL-C, and weight control (26). Thus far, data regarding sex differences in CVD prevention in China are still limited. Future studies are warranted to explore the underlying reasons for sex inequities in medical care.

This study has some potential limitations. First, although a complex sampling framework was used to achieve high representativeness of community-dwelling adults, this study only included 138 urban and rural communities in Guangzhou, the third largest city with developed socioeconomic status in China. Thus, our results cannot be directly generalized to other populations. Second, compared with those excluded due to missing covariates, participants included in the current analysis tended to be more women and older. We cannot completely exclude the possibility of reduced sample representativeness caused by missing data. However, considering the high completeness of collected variables (only 3.6% had missing data in the overall sample), our major results might not be easily affected. Third, information on established ASCVD relied on self-reported and only prior histories of stroke and myocardial infarction were collected, which may result in misclassification bias and overestimating the prevalence of high ASCVD risk. Fourth, although most risk factors used in risk assessment charts were collected by objective measurements, information on smoking status was self-reported, which might cause an underestimation of smoking prevalence as well as ASCVD risk in the population, especially among women. Fifth, although the burden of ASCVD in the current analysis was estimated using a risk prediction chart recommended in the Chinese Guideline for the Prevention of Cardiovascular Disease 2017, the estimated prevalence of high 10-year risk might be sensitive to the risk prediction model used and this could be a possible reason for differences between studies. A recent systematic review suggested that several risk prediction models have been developed among Chinese adults, but direct comparisons and complete external validation for these models are still warranted in further studies (39). Sixth, the association between ASCVD burden and relevant risk factors was evaluated in this cross-sectional study, thus residual confounding and reverse causality might exist.

## Conclusions

In this survey study, the estimated prevalence of 10-year high ASCVD risk was high in Guangzhou, a populated and prosperous city in China. However, the rates of treatment and risk factor control in primary prevention were still far from optimal, especially for lipid management. These findings suggested that substantial improvement in ASCVD prevention is needed in China.

## Data Availability

The datasets presented in this article are not readily available because we prioritises access to data to researchers who have worked on the research study for a substantial duration, have played intellectual and operational roles, and have participated in raising the funds to conduct the study. Data will be disclosed only upon request to the authors and approval of the proposed use of the data by a review committee. Requests to access the datasets should be directed to gzcdc_zhouq@gz.gov.cn.
