# Supplementary material for: Prevalence, awareness, treatment, and risk factor control of high atherosclerotic cardiovascular disease risk in Guangzhou, China
Source: Front Cardiovasc Med. 2023 Jul 14;10:1092058. doi: 10.3389/fcvm.2023.1092058 (PMC10379630; doi:10.3389/fcvm.2023.1092058)
Supplement: Supplementary file 1 [file Table1.docx]

**Supplement Materials**

**Figure S1. Sampling framework of Guangzhou survey in 2018**

| **Flowchart for the risk assessment of atherosclerotic cardiovascular diseases** | | | | |
| --- | --- | --- | --- | --- |
| Those who meet any of the following criteria can be directly classified as high or extremely high risk without the need for ASCVD risk assessment: | | | | |
| Extremely high risk: | | Patients with ASCVD (including those with symptomatic PAD) | | |
| High risk: | | 1. Patients with diabetes (age ≥ 40 years) | | |
|  |  | 2. Individuals with very high levels of a single risk factor, including:  (1) LDL-C ≥ 4.9mmol/L (190mg/dl) or TC ≥ 7.2mmol/L(280mg/dl);  (2) Grade 3 hypertension; (3) Heavy smoking (smoking ≥ 30 cigarettes/d) | | |
| For those who do not meet the conditions, the 10-year risk of developing ASCVD was assessed according to the following table | | | | |
| Number of risk factors ^a^ | | Stratification of serum cholesterol levels (mmol/L) | | |
|  |  | 3.1 ≤ TC < 4.1  or 1.8 ≤ LDL-C < 1.6 | 4.1 ≤ TC < 5.2  or 2.6 ≤ LDL-C < 3.4 | 5.2 ≤ TC < 7.2  or 3.4 ≤ LDL-C < 4.9 |
| Without hypertension | 0-1 | Low risk (<5%) | Low risk (<5%) | Low risk (<5%) |
|  | 2 | Low risk (<5%) | Low risk (<5%) | Moderate risk (5-9%) |
|  | 3 | Low risk (<5%) | Moderate risk (5-9%) | Moderate risk (5-9%) |
| With hypertension | 0 | Low risk (<5%) | Low risk (<5%) | Low risk (<5%) |
|  | 1 | Low risk (<5%) | Moderate risk (5-9%) | Moderate risk (5-9%) |
|  | 2 | Moderate risk (5-9%) | High risk (≥10%) | High risk (≥10%) |
|  | 3 | High risk (≥10%) | High risk (≥10%) | High risk (≥10%) |
| Assessment of lifetime risk for those ten-year ASCVD risk is moderate and aged <55 years. | | | | |
| People with any 2 or more of the following risk factors are defined as being at high risk for ASCVD   - Systolic blood pressure ≥ 160 mmHg or diastolic blood pressure ≥ 100 mmHg - Non-HDL-C ≥ 5.2 mmol/L (200mg/dl) - HDL-C < 1.0mmol/L (40mg/dl) - BMI ≥ 28kg/m^2^ - Smoking | | | | |
| Note. Abbreviation. ASCVD, atherosclerotic cardiovascular disease; PAD: peripheral arterial disease, TC: total cholesterol, LDL-C: low density lipoprotein cholesterol, HDL-C: high density lipoprotein cholesterol, BMI: body mass index. 1 mmHg = 0.133 kPa.  ^a^ Risk factors included smoking, low HDL-C, and being ≥ 45 years old for men or ≥ 55 years old for women. | | | | |

**Figure S2. Flowchart for the risk assessment of atherosclerotic cardiovascular diseases**

**Table S1. Characteristics of the included and excluded samples [n(%)]^a^**

| Characteristic | included samples  (n=15165) | | excluded samples (n=562) | | P value |
| --- | --- | --- | --- | --- | --- |
|  | n | %(95%CI) | n | %(95%CI) |  |
| Sex |  |  |  |  | <0.001 |
| Men | 5962 | 51.9 (49.5, 54.4) | 275 | 63.8 (58.3, 68.9) |  |
| women | 9203 | 48.1 (45.6, 50.5) | 287 | 36.2 (31.1, 41.7) |  |
| Age, year |  |  |  |  | 0.029 |
| 18~<25 | 612 | 12.7 (10.4, 15.4) | 23 | 21.7 (8.4, 45.7) |  |
| 25~<35 | 2133 | 28.1 (24.4, 32.1) | 66 | 24.3 (18.5, 31.3) |  |
| 35~<45 | 2584 | 22.3 (19.9, 24.9) | 78 | 19.0 (11.0, 30.7) |  |
| 45~<55 | 3268 | 19.1 (15.8, 22.9) | 104 | 15.6 (12.2, 19.7) |  |
| 55~<65 | 3851 | 10.3 (9.0, 11.8) | 164 | 11.0 (6.1, 19.1) |  |
| ≥65 | 2717 | 7.5 (6.2, 9.0) | 127 | 8.3 (4.9, 13.9) |  |
| Education level ^b^ |  |  |  |  | 0.684 |
| Primary school or less | 3740 | 13.7 (11.7, 16.1)) | 140 | 12.1 (7.5, 19.0) |  |
| Middle school/High school | 8160 | 51.6 (47.1, 56.0) | 286 | 48.1 (38.5, 57.8) |  |
| College or higher | 3265 | 34.7 (29.1, 40.7) | 130 | 39.8 (27.9, 53.0) |  |
| Residence |  |  |  |  | 0.276 |
| Urban | 10739 | 68.3 (55.5, 78.9) | 398 | 77.6 (57.4, 89.9) |  |
| Rural | 4426 | 31.7 (21.1, 44.5) | 164 | 22.4 (10.1, 42.6) |  |

Note. Abbreviation. CIs, confidence intervals. a No. of participants was the unweighted number of subcategories denominator; the percentages were weighted. b Six missing values.

**Table S2. The distribution of lifetime ASCVD risk [n(%)] ^a^**

| Characteristic | Participants without high risk of lifetime ASCVD | | Participants with high risk of lifetime ASCVD | |
| --- | --- | --- | --- | --- |
|  | n | %(95%CI) | n | %(95%CI) |
|  | 242 | 53.7(47.1,60.1) | 173 | 46.3(39.9,52.9) |
| Sex |  |  |  |  |
| Men | 231 | 53.2(46.6,59.7) | 165 | 46.8(40.3,53.4) |
| women | 11 | 70.4(35.0,91.3) | 8 | 29.6(8.7,65.0) |
| Age, year |  |  |  |  |
| 18~<25 | 1 | 18.3(2.3,67.7) | 5 | 81.7(32.3,97.7) |
| 25~<35 | 11 | 14.6(6.5,29.7) | 38 | 85.4(70.3,93.5) |
| 35~<45 | 27 | 34.8(22.9,48.9) | 51 | 65.2(51.1,77.1) |
| 45~<55 | 203 | 74.7(68.0,80.5) | 79 | 25.3(19.5,32.0) |
| Education level |  |  |  |  |
| Primary school or less | 42 | 68.6(47.2,84.2) | 13 | 31.4(15.8,52.8) |
| Middle school/High school | 164 | 55.5(48.6,62.2) | 122 | 44.5(37.8,51.4) |
| College or higher | 36 | 41.4(24.6,60.4) | 38 | 58.6(39.6,75.4) |
| Residence |  |  |  |  |
| Urban | 142 | 55.6(46.5,64.4) | 98 | 44.4(35.6,53.5) |
| Rural | 100 | 51.2(43.0,59.3) | 75 | 48.8(40.7,57.0) |

Note. Abbreviation. ASCVD, atherosclerotic cardiovascular disease; CIs, confidence intervals. a No. of participants was the unweighted number of subcategories denominator; the percentages were weighted.

**Table S3. Primary and secondary prevention for CVD a**

|  | Participants with high10-year risk of ASCVD [N=3075] | | | | with established CVD[N=289] | | | |
| --- | --- | --- | --- | --- | --- | --- | --- | --- |
|  | n | % (95%CIs) | p Value d | OR (95%CIs) | n | % (95%CIs) | p Value d | OR (95%CIs) |
| **Medication use** |  |  |  |  |  |  |  |  |
| BP lowering drugs (%) |  |  |  |  |  |  |  |  |
| Overall | 890 | 37.7 (32.8-42.8) |  |  | 163 | 75.9 (64.3-84.7) |  |  |
| Sex |  |  | 0.028 |  |  |  | 0.454 |  |
| Men | 463 | 31.3 (27.0-35.9) |  | 1 (Ref) | 95 | 80.5 (67.9-89.0) |  | 1 (Ref) |
| Women | 427 | 51.8 (44.3-59.3) |  | 1.58 (1.05-2.38) | 68 | 67.4 (52.3-79.6) |  | 0.74 (0.33-1.65) |
| Residence |  |  | 0.018 |  |  |  | 0.099 |  |
| Urban | 608 | 41.4 (34.2-49.0) |  | 1.54 (1.08-2.21) | 131 | 79.7(64.8-89.3) |  | 2.54 (0.83-7.71) |
| Rural | 282 | 31.6 (26.9-36.8) |  | 1 (Ref) | 32 | 60.8 (41.7-77.0) |  | 1 (Ref) |
| Lipid-lowering drugs (%) |  |  |  |  |  |  |  |  |
| Overall | 174 | 6.7 (4.5-10.0) |  |  | 57 | 42.4 (29.5-56.4) |  |  |
| Sex |  |  | 0.164 |  |  |  | 0.081 |  |
| Men | 81 | 5.2 (3.4-8.0) |  | 1 (Ref) | 33 | 51.1 (32.3-69.7) |  | 1 (Ref) |
| Women | 93 | 9.7 (6.2-14.8) |  | 1.71 (0.80-3.67) | 24 | 29.2 (19.3-41.6) |  | 0.42 (0.16-1.12) |
| Residence |  |  | 0.049 |  |  |  | 0.069 |  |
| Urban | 147 | 8.6 (5.5-13.3) |  | 2.18 (1.00-4.73) | 51 | 48.1 (32.5-64.0) |  | 3.76 (0.90-15.75) |
| Rural | 27 | 3.2 (1.8-5.5) |  | 1 (Ref) | 6 | 19.4 (8.6-38.1) |  | 1 (Ref) |
| Glucose-lowering medications (%) |  |  |  |  |  |  |  |  |
| Overall | 734 | 41.4 (35.8-47.3) |  |  | 52 | 62.0 (47.5-76.4) |  |  |
| Sex |  |  | 0.218 |  |  |  | 0.030 |  |
| Men | 307 | 36.6 (30.8-42.9) |  | 1 (Ref) | 28 | 55.8 (36.0-73.8) |  | 1 (Ref) |
| Women | 427 | 47.5 (41.0-54.2) |  | 1.21 (0.89-1.64) | 24 | 77.1 (55.3-90.2) |  | 5.62 (1.19-26.57) |
| Residence |  |  | <0.001 |  |  |  | 0.765 |  |
| Urban | 559 | 45.2 (38.6-52.0) |  | 2.13 (1.42-3.19) | 43 | 64.4 (47.6-78.2) |  | 1.23 (0.31-4.92) |
| Rural | 175 | 30.2 (23.8-37.5) |  | 1 (Ref) | 9 | 52.0 (24.7-78.1) |  | 1 (Ref) |
| Aspirin (%) |  |  |  |  |  |  |  |  |
| Overall | 54 | 1.0 (0.6-1.8) |  |  | 55 | 21.3 (13.8-31.5) |  |  |
| Sex |  |  | 0.252 |  |  |  | 0.450 |  |
| Men | 28 | 0.7 (0.3-1.3) |  | 1 (Ref) | 35 | 20.2 (11.9-32.3) |  | 1 (Ref) |
| Women | 26 | 1.8 (0.9-3.4) |  | 1.59 (0.71-3.55) | 20 | 23.8 (13.5-38.3) |  | 1.38 (0.59-3.23) |
| Residence |  |  | 0.014 |  |  |  | 0.003 |  |
| Urban | 48 | 1.5 (0.9-2.6) |  | 6.05 (1.46-25.03) | 53 | 26.1 (17.3-37.5) |  | 12.15 (2.49-59.21) |
| Rural | 6 | 0.2 (0.0-0.8) |  | 1 (Ref) | 2 | 2.5 (0.5-11.1) |  | 1 (Ref) |
| Statins (%) |  |  |  |  |  |  |  |  |
| Overall | 70 | 1.7 (1.0-3.1) |  |  | 72 | 29.4 (21.2-39.1) |  |  |
| Sex |  |  | 0.099 |  |  |  | 0.566 |  |
| Men | 30 | 1.1 (0.6-2.1) |  | 1 (Ref) | 41 | 29.2 (18.7-42.6) |  | 1 (Ref) |
| Women | 40 | 3.1 (1.5-6.2) |  | 2.43 (0.84-7.02) | 31 | 29.7 (19.6-42.4) |  | 1.29 (0.54-3.11) |
| Residence |  |  | 0.051 |  |  |  | 0.125 |  |
| Urban | 60 | 2.4 (1.3-4.5) |  | 2.86 (1.00-8.23) | 66 | 33.5 (24.1-44.3) |  | 3.30 (0.71-15.33) |
| Rural | 10 | 0.6 (0.2-1.3) |  | 1 (Ref) | 6 | 13.4 (3.7-38.3) |  | 1 (Ref) |
| **Risk factor control** |  |  |  |  |  |  |  |  |
| BP<130/80 |  |  |  |  |  |  |  |  |
| Overall | 861 | 29.3 (25.7-33.2) |  |  | 79 | 28.1 (19.8-38.2) |  |  |
| Sex |  |  | <0.001 |  |  |  | 0.757 |  |
| Men | 379 | 26.4 (22.8-30.4) |  | 1 (Ref) | 45 | 29.4 (20.3-40.6) |  | 1 (Ref) |
| Women | 482 | 35.3 (29.6-41.5) |  | 1.69 (1.33-2.14) | 34 | 25.2 (13.1-43.1) |  | 0.87 (0.35-2.13) |
| Residence |  |  | 0.004 |  |  |  | 0.053 |  |
| Urban | 668 | 32.8 (27.8-38.2) |  | 1.61 (1.17-2.21) | 69 | 30.7 (20.6-43.0) |  | 3.34 (0.98-11.36) |
| Rural | 193 | 23.0 (20.1-26.2) |  | 1 (Ref) | 10 | 18.1 (8.8-33.6) |  | 1 (Ref) |
| LDL-C<2.6 |  |  |  |  |  |  |  |  |
| Overall | 569 | 16.8 (15.0-18.6) |  |  | 134 | 48.5 (40.4-56.6) |  |  |
| Sex |  |  | 0.773 |  |  |  | 0.001 |  |
| Men | 352 | 16.8 (14.2-19.6) |  | 1 (Ref) | 94 | 58.7 (48.6-68.1) |  | 1 (Ref) |
| Women | 217 | 16.8 (13.8-20.3) |  | 0.92 (0.58-1.47) | 40 | 25.9 (17.6-36.4) |  | 0.26 (0.12-0.56) |
| Residence |  |  | 0.242 |  |  |  | 0.001 |  |
| Urban | 376 | 16.1 (14.3-18.2) |  | 0.84 (0.63-1.13) | 113 | 54.1 (44.5-63.5) |  | 3.48 (1.69-7.13) |
| Rural | 193 | 17.9 (14.7-21.6) |  | 1 (Ref) | 21 | 26.4 (17.2-38.3) |  | 1 (Ref) |
| Normal weight b |  |  |  |  |  |  |  |  |
| Overall | 1233 | 36.0 (31.1-41.2) |  |  | 124 | 40.5 (33.3-48.2) |  |  |
| Sex |  |  | 0.117 |  |  |  | 0.563 |  |
| Men | 638 | 33.7 (28.1-39.7) |  | 1 (Ref) | 75 | 41.1 (32.1-50.8) |  | 1 (Ref) |
| Women | 595 | 40.8 (35.9-45.8) |  | 1.23 (0.95-1.61) | 49 | 39.2 (27.9-51.9) |  | 1.21 (0.63-2.32) |
| Residence |  |  | 0.554 |  |  |  | 0.033 |  |
| Urban | 841 | 37.8 (31.0-45.1) |  | 1.13 (0.75-1.72) | 88 | 37.4 (29.6-45.8) |  | 0.36 (0.14-0.92) |
| Rural | 392 | 32.8 (27.6-38.4) |  | 1 (Ref) | 36 | 52.9 (36.2-69.0) |  | 1 (Ref) |
| Not a current smoker |  |  |  |  |  |  |  |  |
| Overall | 2002 | 55.8 (50.6-60.9) |  |  | 227 | 76.8 (68.3-83.6) |  |  |
| Sex |  |  | <0.001 |  |  |  | <0.001 |  |
| Men | 623 | 35.5 (30.4-41.0) |  | 1 (Ref) | 117 | 66.3 (55.6-75.7) |  | 1 (Ref) |
| Women | 1379 | 97.4 (95.9-98.4) |  | 66.53 (39.43-112.26) | 110 | 99.9 (99.5-100.0) |  | 816.57 (98.43-6774.29) |
| Residence |  |  | 0.006 |  |  |  | 0.048 |  |
| Urban | 1476 | 64.1 (58.2-69.7) |  | 2.11 (1.25-3.58) | 181 | 78.5 (68.5-86.0) |  | 2.62 (1.01-6.80) |
| Rural | 526 | 40.5 (31.9-49.7) |  | 1 (Ref) | 46 | 70.0 (56.3-80.8) |  | 1 (Ref) |
| Achieving physical activity targets c |  |  |  |  |  |  |  |  |
| Overall | 2308 | 75.4 (71.7-78.7) |  |  | 220 | 73.6 (63.8-81.5) |  |  |
| Sex |  |  | 0.044 |  |  |  | 0.684 |  |
| Men | 1188 | 73.8 (70.0-77.3) |  | 1 (Ref) | 134 | 73.4 (63.3-81.5) |  | 1 (Ref) |
| Women | 1120 | 78.6 (73.4-83.0) |  | 1.30 (1.01-1.68) | 86 | 74.0 (56.6-86.2) |  | 1.17 (0.53-2.61) |
| Residence |  |  | 0.030 |  |  |  | 0.022 |  |
| Urban | 1575 | 77.3 (72.5-81.5) |  | 1.39 (1.03-1.88) | 175 | 77.7 (66.8-85.8) |  | 2.75 (1.16-6.51) |
| Rural | 733 | 71.7 (67.9-75.3) |  | 1 (Ref) | 45 | 57.4 (41.8-71.6) |  | 1 (Ref) |

Note. Abbreviation. ASCVD, atherosclerotic cardiovascular disease; CVD, cardiovascular diseases; established CVD, including stroke, myocardial infarction, angina pectoris, heart bypass surgery, heart stent surgery. a n represents the actual number of people surveyed, and the composition ratio is weighted according to the complex sampling design (%). The use of BP lowering drugs, lipid-lowering drugs and glucose-lowering medications was calculated based on people with hypertension, diabetes and dyslipidaemia respectively; the use of other medicine and the control of risk factors were based on the whole population. b Normal weight means BMI >=18.5~<24.0. c Achieving physical activity targets means >=150 minutes of moderate intensity exercise per week or >=75 minutes of high intensity exercise per week. d P-values and OR (95%CIs) were calculated in logistic regression models, in which the dependent variable was medication use or risk factor control, and the dependent variables included sex, residence, age, and education level
